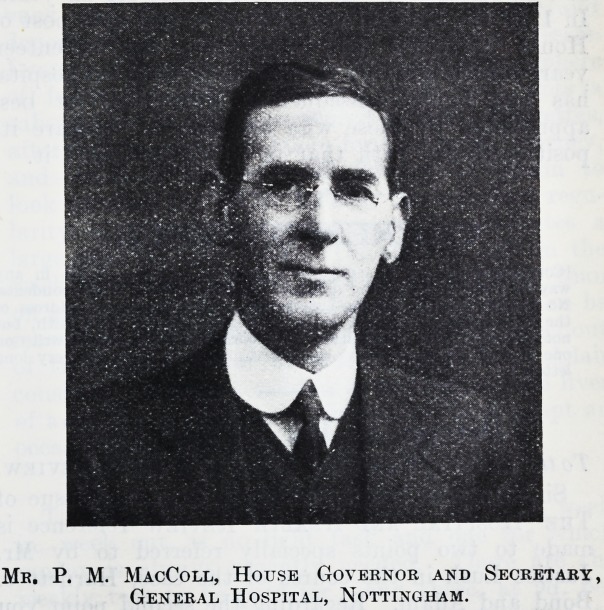# Hospital Men of Mark: Mr. Frederick Action and Mr. Peter McColl

**Published:** 1924-05

**Authors:** 


### HOSPITAL MEN OF MARK.

### MR. FREDERICK ACTON, C.B.E., AND MR. PETER M. MacCOLL.

A particularly active Chairman in the hospital world is Mr. Frederick Acton, C.B.E., of the General Hospital, Nottingham. It was as far back as 1892 that he was first appointed a member of the Board, and in 1894 he was elected Vice-Chairman. From this post he retired two years later in favour of another member, but continued his active services on the Board, and when the late Sir Charles Seely, Bt., retired, after a chairmanship of 17 years, Mr. Acton was unanimously elected to succeed him. When he first became a member of the Committee the Hospital contained 198 beds only, but in 1897 his services were particularly and successfully devoted to the raising of large funds, as the result of which a new wing, consisting of circular wards containing in all 66 beds, was built to commemorate Queen Victoria's Jubilee. In the early days of the war, commodious temporary buildings were erected and more than 6,000 sick and wounded soldiers passed through the Institution between 1914 and 1918, and almost every new arrival was personally received by the Chairman.

[*Short, Nottingham.*

Mr. Frederick Acton, C.B.E., Chairman of the General Hospital, Nottingham.

There are now 310 beds in the Hospital, including a children's ward of 30 beds, while there are 44 beds at The Cedars Convalescent Hospital, situated at Woodthorpe, in beautiful surroundings some miles from the centre of the city, which was presented to the Hospital by the late Sir Charles Seely, and endowed by a gift of £50,000 from Sir Jesse Boot. Within recent years £100,000 was raised by public subscription, the main purpose of which was the provision of a new and much needed Home for the Nursing Staff, to be recognised as a Memorial Home of the City and County of Nottingham, which was opened by the Prince of Wales last August. There are rooms for 130 nurses, lecture and recreation rooms, etc. The money raised was also used for a much needed modernising of the warming of the hospital. The great increase in the work of the Out-Patient Department has necessitated extension in this direction, and steps are now being taken to provide new buildings, including an adequate Out-Patient Department, X-Ray, Ear, Nose and Throat, Orthopædic and Pathological Departments, on a site adjoining the hospital, presented to the institution by the Nottingham Corporation.

It would be difficult to over-estimate the services which Mr. Acton has given to the hospital during a period extending over 32 years. His sound business judgment and particularly his vast experience in legal matters have been of the greatest possible value, and have at all times been given most willingly, the professional services of his office having been rendered throughout without charge. Among his many activities (and he is an extremely busy man, as the senior partner in a well-known firm of solicitors in the city), Mr. Acton holds office as Chairman of the Local Voluntary Hospitals Committee, Chairman of the East Midlands Branch of the British Hospitals Association, President of the Nottingham and Notts. Hospital Saturday Fund, and Deputy-Chairman of the Lindsey Quarter Sessions, Lincs., Vice-Chairman of Ellerslie House (Home for Paralysed Sailors and Soldiers). He is also a Justice of the Peace for Nottinghamshire and Lincolnshire, was High Sheriff of the latter county in 1915-16, and in 1912 succeeded the late Duke of Northumberland as Hon. President of the National Deposit Friendly Society, which post he still holds. He is nearly 80 years of age, and he and Mrs. Acton will, it is hoped, shortly celebrate their diamond wedding.

Mr. P. M. MacColl, House Governor and Secretary, General Hospital, Nottingham.

Mr. Peter M. MacColl, the House Governor and Secretary, has been associated with the hospital for seventeen years. He is a native of Perthshire and was educated in Greenock. He came south in 1892, and after spending several years in the commercial world, became a member of the staff of Messrs. Frank Impey and Co., a well-known firm of chartered accountants in Birmingham, where he remained eleven years. During that time he not only had ample opportunities of gaining experience in accountancy, but acquired a wide knowledge of secretarial work in connection with various organisations, including the old Bedstead Manufacturers' Association, the Birmingham Rowton Houses, Limited, and the Birmingham Hospital Sunday Fund. In 1907 he was appointed Assistant Secretary at the Nottingham General Hospital from among 198 candidates, and in 1918 was appointed Secretary in succession to Mr. E. M. Keely, who had held office for 32 years. In 1919 Mr. MacColl was elected to the dual post of House Governor and Secretary. His seventeen years' association with the management of the hospital has had happy consequences which can be best appreciated by those who are able to compare its position to-day with that in which he found it.

## Figures and Tables

**Figure f1:**
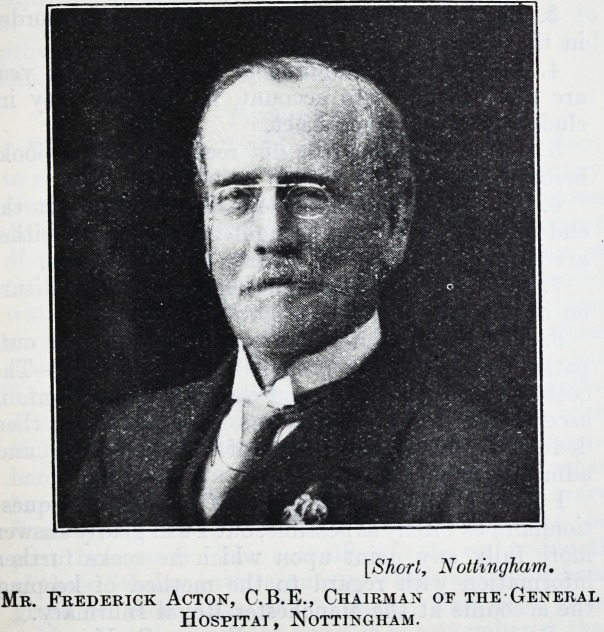


**Figure f2:**